# Supplementary material for: Baseline associations between household air pollution exposure and blood pressure among pregnant women in the Household Air Pollution Intervention Network (HAPIN) multi-country randomized controlled trial
Source: medRxiv. 2023 Jan 26:2023.01.23.23284847. Preprint. [Version 1] doi: 10.1101/2023.01.23.23284847 (PMC9901046; doi:10.1101/2023.01.23.23284847)
Supplement: Supplement 1 [file media-1.pdf]

Baseline associations between household air pollution exposure and blood pressure among pregnant women in the Household Air Pollution Intervention Network (HAPIN) multi-country randomized controlled trial

Table of Contents

**Table S1**. Measured 24-hour personal exposures to $PM_{2.5}$, BC, and CO by IRC, after removing the lowest and the highest 2.5% of exposure samples	2
**Table S2.** Trial-wide adjusted association between HAP exposure and PP/MAP	2
**Table S3a.** Adjusted association between HAP and SBP/DBP in Guatemala	3
**Table S3b.** Adjusted association between HAP and PP/MAP in Guatemala	4
**Table S4a.** Adjusted association between HAP and SBP/DBP in India	5
**Table S4b.** Adjusted association between HAP and PP/MAP in India	6
**Table S5a.** Adjusted association between HAP and SBP/DBP in Peru	7
**Table S5b.** Adjusted association between HAP and PP/MAP in Peru	8
**Table S6a.** Adjusted association between HAP and SBP/DBP in Rwanda	9
**Table S6b.** Adjusted association between HAP and PP/MAP in Rwanda	10
**Table S7.** Trial-wide association between HAP exposure and blood pressure parameters (without the highest and lowest 2.5% of the exposure samples)	11
**Table S8.** Effect modification for the HAP-BP association in Guatemala	11
**Table S9.** Effect modification for the HAP-BP association in India	13
**Table S10.** Effect modification for the HAP-BP association in Peru	14
**Table S11.** Effect modification for the HAP-BP association in Rwanda	15
**Table S12**. Summary of SBP, DBP and selected baseline characteristics in population with and without missing exposures to $PM_{2.5}$, BC, and CO	16
**Figure S1**. Directed Acyclic Graph (DAG) for the HAP-BP association	17
**Figure S2.** Distributions of gestational age at baseline	18
**Figure S3.** Distributions of systolic (top) and diastolic (bottom) blood pressure at baseline	19
**Figure S4.** HAP-GBP association and 95% confidence intervals in Guatemala	20
**Figure S5.** HAP-GBP association and 95% confidence intervals in India	21
**Figure S6.** HAP-GBP association and 95% confidence intervals in Peru	22
**Figure S7.** HAP-GBP association and 95% confidence intervals in Rwanda	23

Table S1. Measured 24-hour personal exposures to $PM_{2.5}$, BC, and CO by IRC, after removing the lowest and the highest 2.5% of exposure samples

	N	Median	IQR	Mean	Min	Max
PM2.5 > 14.25 and PM2.5 < 401.55 (removed highest and lowest 2.5%)						
All IRCs	2676	82.85	93.75	104.80	14.25	400.05
Guatemala	699	110.40	114.00	130.40	15.13	392.30
India	685	75.08	78.85	97.94	15.85	388.60
Peru	591	54.94	89.24	82.63	14.25	378.50
Rwanda	701	88.35	81.26	104.70	14.29	400.10
BC > 1.57 and BC < 39.30 (removed highest and lowest 2.5%)						
All IRCs	2408	10.69	8.20	11.61	1.57	38.83
Guatemala	665	11.90	5.70	12.37	2.55	34.30
India	656	9.41	9.94	11.72	1.58	38.39
Peru	531	8.72	11.37	10.69	1.57	38.83
Rwanda	556	10.87	7.42	11.46	2.66	35.97
CO > 0.03 and CO < 13.63 (removed highest and lowest 2.5%)						
All IRCs	2728	1.24	2.13	2.03	0.03	13.63
Guatemala	741	1.36	2.09	1.94	0.03	12.61
India	697	0.88	1.73	1.58	0.03	12.15
Peru	616	1.81	3.33	2.84	0.03	13.63
Rwanda	674	1.08	1.71	1.86	0.03	13.56

Table S2. Trial-wide adjusted association between HAP exposure and PP/MAP.

	$PM_{2.5}$		BC		CO	
	Estimate	95% CI	Estimate	95% CI	Estimate	95% CI
Pulse Pressure (PP)						
Log linear	0.32	(-0.05, 0.70)	0.58	(-0.14, 1.29)	0.03	(-0.15, 0.21)
Categorical [Ref. Quartile 1]						
Quartile 2	0.07	(-0.64, 0.78)	-0.34	(-1.09, 0.41)	0.13	(-0.56, 0.83)
Quartile 3	0.49	(-0.42, 1.41)	0.21	(-0.62, 1.05)	0.73	(0.03, 1.42)
Quartile 4	0.52	(-0.20, 1.25)	1.04	(-0.06, 2.14)	-0.19	(-0.89, 0.52)
Mean Arterial Pressure (MAP)						
Log linear	-0.18	(-0.71, 0.36)	-0.07	(-0.59, 0.45)	-0.01	(-0.34, 0.33)
Categorical [Ref. Quartile 1]						
Quartile 2	-0.12	(-0.86, 0.63)	-0.45	(-1.23, 0.33)	-0.58	(-2.20, 1.03)
Quartile 3	-0.25	(-1.37, 0.87)	-0.52	(-1.94, 0.91)	0.18	(-0.96, 1.32)
Quartile 4	-0.05	(-1.14, 1.03)	-0.35	(-1.63, 0.93)	-0.15	(-1.62, 1.32)

Note:

1. All models controlled for gestational age at the BP measurement, BMI, and mother's age. Additional covariates are controlled for IRC-specific models (Guatemala: nulliparity, mother's education, physical activity, date of the BP measurement, mother's diet diversity and season; India: mother's education, household wealth, and season; Peru: physical activity, time of the BP measurement, household food insecurity, mother's diet diversity and season; Rwanda: nulliparity, mother's education, physical activity, time of the BP measurement, household food insecurity and smoker at home)

2. In log linear models, the coefficients indicate the increase in BP (mmHg) per a one unit increase in the log of exposure.

3. Shaded cells are fixed effects, unshaded are random effects, meta-analyses combining results across 4 IRCs

Table S3a. Adjusted association between HAP and SBP/DBP in Guatemala

	Model Type	Estimate	p-value	95% CI	AIC
Systolic Blood Pressure					
PM2.5	Linear	3.00E-04	0.9069	(-0.0046, 0.0051)	5139
	Log linear	-0.0115	0.9776	(-0.8122, 0.7892)	5139
	Categorical [Ref. Quartile 1)				
	Quartile 2	0.7817	0.3727	(-0.9388, 2.5022)	5142
	Quartile 3	0.4535	0.6087	(-1.2849, 2.192)	5142
	Quartile 4	0.5567	0.5318	(-1.1906, 2.3039)	5142
BC	Linear	0.028	0.4141	(-0.0392, 0.0952)	4700
	Log linear	0.4565	0.4917	(-0.8464, 1.7593)	4700
	Categorical [Ref. Quartile 1)				
	Quartile 2	-0.2543	0.7779	(-2.024, 1.5154)	4704
	Quartile 3	-0.1518	0.8662	(-1.9199, 1.6164)	4704
	Quartile 4	0.5654	0.5313	(-1.207, 2.3379)	4704
CO	Linear	-0.0378	0.702	(-0.2316, 0.156)	5245
	Log linear	-0.1647	0.5092	(-0.6544, 0.325)	5245
	Categorical [Ref. Quartile 1)				
	Quartile 2	-2.0871	0.0153	(-3.7726, -0.4015)	5242
	Quartile 3	-0.454	0.5999	(-2.1526, 1.2446)	5242
	Quartile 4	-0.9296	0.2891	(-2.6501, 0.7909)	5242
Diastolic Blood Pressure					
PM2.5	Linear	-0.001	0.6146	(-0.0051, 0.003)	4882
	Log linear	-0.2189	0.5221	(-0.8899, 0.4521)	4882
	Categorical [Ref. Quartile 1)				
	Quartile 2	0.6895	0.348	(-0.752, 2.131)	4885
	Quartile 3	0.2093	0.778	(-1.2473, 1.6658)	4885
	Quartile 4	-0.1483	0.8424	(-1.6123, 1.3156)	4885
BC	Linear	0.0169	0.555	(-0.0393, 0.0731)	4461
	Log linear	-0.0319	0.9542	(-1.121, 1.0573)	4461
	Categorical [Ref. Quartile 1)				
	Quartile 2	-0.0011	0.9989	(-1.4807, 1.4785)	4465
	Quartile 3	-0.112	0.8818	(-1.5902, 1.3663)	4465
	Quartile 4	-0.417	0.5808	(-1.8989, 1.0649)	4465
CO	Linear	-0.062	0.4593	(-0.2264, 0.1024)	5001
	Log linear	-0.1775	0.4018	(-0.5929, 0.2379)	5001
	Categorical [Ref. Quartile 1)				
	Quartile 2	-1.5119	0.0386	(-2.9443, -0.0796)	5001
	Quartile 3	-0.7466	0.3103	(-2.19, 0.6969)	5001
	Quartile 4	-0.8224	0.2698	(-2.2845, 0.6397)	5001

Note: *All models controlled for gestational age at the BP measurement, BMI, and mother's age, nulliparity, mother's education, physical activity, date of the BP measurement, mother's diet diversity and season*

Table S3b. Adjusted association between HAP and PP/MAP in Guatemala

	Model Type	Estimate	p-value	95% CI	AIC
Pulse Pressure					
PM2.5	Linear	0.0013	0.4410	(-0.0021, 0.0047)	4620
	Log linear	0.2074	0.4673	(-0.3526, 0.7674)	4620
	Categorical [Ref. Quartile 1)				
	Quartile 2	0.0922	0.8804	(-1.111, 1.2954)	4623
	Quartile 3	0.2443	0.6934	(-0.9715, 1.46)	4623
	Quartile 4	0.7050	0.2577	(-0.5169, 1.9269)	4623
BC	Linear	0.0111	0.6536	(-0.0373, 0.0595)	4263
	Log linear	0.4883	0.3071	(-0.4498, 1.4264)	4262
	Categorical [Ref. Quartile 1)				
	Quartile 2	-0.2532	0.6959	(-1.5248, 1.0184)	4263
	Quartile 3	-0.0398	0.9510	(-1.3102, 1.2306)	4263
	Quartile 4	0.9824	0.1303	(-0.2911, 2.2559)	4263
CO	Linear	0.0242	0.7260	(-0.1114, 0.1598)	4715
	Log linear	0.0128	0.9416	(-0.3299, 0.3555)	4715
	Categorical [Ref. Quartile 1)				
	Quartile 2	-0.5752	0.3401	(-1.758, 0.6077)	4717
	Quartile 3	0.2926	0.6301	(-0.8994, 1.4846)	4717
	Quartile 4	-0.1072	0.8616	(-1.3146, 1.1001)	4717
Mean Arterial Pressure					
PM2.5	Linear	-0.0006	0.7709	(-0.0046, 0.0034)	4873
	Log linear	-0.1497	0.6593	(-0.8162, 0.5167)	4873
	Categorical [Ref. Quartile 1)				
	Quartile 2	0.7203	0.3237	(-0.7116, 2.1521)	4876
	Quartile 3	0.2907	0.6934	(-1.1561, 1.7375)	4876
	Quartile 4	0.0867	0.9069	(-1.3675, 1.5408)	4876
BC	Linear	0.0206	0.4672	(-0.035, 0.0761)	4447
	Log linear	0.1309	0.8115	(-0.9466, 1.2085)	4447
	Categorical [Ref. Quartile 1)				
	Quartile 2	-0.0855	0.9088	(-1.5498, 1.3788)	4451
	Quartile 3	-0.1252	0.8666	(-1.5882, 1.3378)	4451
	Quartile 4	-0.0895	0.9046	(-1.5561, 1.377)	4451
CO	Linear	-0.0539	0.5152	(-0.2166, 0.1087)	4985
	Log linear	-0.1733	0.4081	(-0.5842, 0.2377)	4985
	Categorical [Ref. Quartile 1)				
	Quartile 2	-1.7036	0.0184	(-3.1192, -0.2881)	4983
	Quartile 3	-0.6490	0.3720	(-2.0756, 0.7775)	4983
	Quartile 4	-0.8581	0.2440	(-2.3031, 0.5868)	4983

Note: *All models controlled for gestational age at the BP measurement, BMI, and mother's age, nulliparity, mother's education, physical activity, date of the BP measurement, mother's diet diversity and season.*

Table S4a. Adjusted association between HAP and SBP/DBP in India

	Model Type	Estimate	p-value	95% CI	AIC
Systolic Blood Pressure					
PM2.5	Linear	0.0023	0.3281	(-0.0023, 0.0069)	5177
	Log linear	0.4411	0.2985	(-0.3913, 1.2734)	5177
	Categorical [Ref. Quartile 1)				
	Quartile 2	-0.0261	0.9785	(-1.9248, 1.8726)	5181
	Quartile 3	0.5091	0.601	(-1.4014, 2.4196)	5181
	Quartile 4	0.9729	0.3173	(-0.9358, 2.8816)	5181
BC	Linear	0.0284	0.3559	(-0.032, 0.0888)	5066
	Log linear	0.33	0.4392	(-0.507, 1.167)	5066
	Categorical [Ref. Quartile 1)				
	Quartile 2	0.3304	0.7338	(-1.5764, 2.2371)	5068
	Quartile 3	1.0504	0.2849	(-0.8766, 2.9775)	5068
	Quartile 4	1.1592	0.242	(-0.7845, 3.1028)	5068
CO	Linear	0.0874	0.3966	(-0.115, 0.2898)	5261
	Log linear	0.3903	0.0604	(-0.0171, 0.7976)	5259
	Categorical [Ref. Quartile 1)				
	Quartile 2	1.2883	0.1728	(-0.5651, 3.1417)	5262
	Quartile 3	1.9096	0.042	(0.0688, 3.7504)	5262
	Quartile 4	1.0558	0.2642	(-0.7993, 2.9108)	5262
Diastolic Blood Pressure					
PM2.5	Linear	0.0023	0.231	(-0.0015, 0.006)	4882
	Log linear	0.6528	0.0584	(-0.0231, 1.3287)	4879
	Categorical [Ref. Quartile 1)				
	Quartile 2	-0.3217	0.6812	(-1.8585, 1.2151)	4878
	Quartile 3	1.2127	0.1241	(-0.3337, 2.7591)	4878
	Quartile 4	1.6491	0.0365	(0.1042, 3.194)	4878
BC	Linear	0.0271	0.282	(-0.0223, 0.0765)	4785
	Log linear	0.5038	0.1487	(-0.1804, 1.1881)	4784
	Categorical [Ref. Quartile 1)				
	Quartile 2	0.2138	0.7876	(-1.344, 1.7717)	4786
	Quartile 3	1.2862	0.1092	(-0.2882, 2.8606)	4786
	Quartile 4	1.2893	0.1114	(-0.2987, 2.8773)	4786
CO	Linear	0.0833	0.3292	(-0.0842, 0.2507)	4985
	Log linear	0.3595	0.0365	(0.0226, 0.6963)	4981
	Categorical [Ref. Quartile 1)				
	Quartile 2	0.7423	0.3412	(-0.7878, 2.2724)	4982
	Quartile 3	1.5339	0.0479	(0.0141, 3.0536)	4982
	Quartile 4	1.9873	0.0111	(0.4558, 3.5187)	4982

Note: *All models controlled for gestational age at baseline, BMI, and mother's age, mother's education, household wealth, and season.*

Table S4b. Adjusted association between HAP and PP/MAP in India

	Model Type	Estimate	p-value	95% CI	AIC
Pulse Pressure					
PM2.5	Linear	0	0.9965	(-0.0038, 0.0038)	4911
	Log linear	-0.2117	0.5476	(-0.9027, 0.4793)	4911
	Categorical [Ref. Quartile 1)				
	Quartile 2	0.2956	0.7125	(-1.2789, 1.8701)	4913
	Quartile 3	-0.7036	0.3835	(-2.2879, 0.8807)	4913
	Quartile 4	-0.6762	0.4019	(-2.259, 0.9066)	4913
BC	Linear	0.0013	0.9586	(-0.0486, 0.0512)	4799
	Log linear	-0.1739	0.6216	(-0.8652, 0.5175)	4799
	Categorical [Ref. Quartile 1)				
	Quartile 2	0.1165	0.8846	(-1.46, 1.6931)	4803
	Quartile 3	-0.2358	0.7715	(-1.829, 1.3575)	4803
	Quartile 4	-0.1302	0.8737	(-1.7372, 1.4769)	4803
CO	Linear	0.0042	0.9606	(-0.1612, 0.1695)	4966
	Log linear	0.0308	0.8562	(-0.3027, 0.3642)	4966
	Categorical [Ref. Quartile 1)				
	Quartile 2	0.546	0.4789	(-0.9671, 2.0591)	4966
	Quartile 3	0.3758	0.6236	(-1.127, 1.8786)	4966
	Quartile 4	-0.9315	0.2276	(-2.4459, 0.5829)	4966
Mean Arterial Pressure					
PM2.5	Linear	0.0023	0.2158	(-0.0013, 0.0059)	4837
	Log linear	0.5822	0.0815	(-0.0731, 1.2375)	4835
	Categorical [Ref. Quartile 1)				
	Quartile 2	-0.2232	0.7691	(-1.7149, 1.2686)	4836
	Quartile 3	0.9782	0.2012	(-0.5229, 2.4792)	4836
	Quartile 4	1.4237	0.0627	(-0.0759, 2.9233)	4836
BC	Linear	0.0275	0.259	(-0.0203, 0.0754)	4740
	Log linear	0.4459	0.1871	(-0.217, 1.1088)	4740
	Categorical [Ref. Quartile 1)				
	Quartile 2	0.2527	0.7424	(-1.2564, 1.7618)	4742
	Quartile 3	1.2076	0.1205	(-0.3176, 2.7328)	4742
	Quartile 4	1.2459	0.1122	(-0.2924, 2.7843)	4742
CO	Linear	0.0847	0.3054	(-0.0774, 0.2468)	4937
	Log linear	0.3697	0.0263	(0.0438, 0.6957)	4933
	Categorical [Ref. Quartile 1)				
	Quartile 2	0.9243	0.2213	(-0.558, 2.4066)	4936
	Quartile 3	1.6591	0.0272	(0.1869, 3.1313)	4936
	Quartile 4	1.6768	0.0268	(0.1932, 3.1603)	4936

Note: *All models controlled for gestational age at baseline, BMI, and mother's age, mother's education, household wealth, and season.*

Table S5a. Adjusted association between HAP and SBP/DBP in Peru

	Model Type	Estimate	p-value	95% CI	AIC
Systolic Blood Pressure					
PM2.5	Linear	0.0014	0.6444	(-0.0044, 0.0072)	4384
	Log linear	-0.0235	0.9406	(-0.6412, 0.5943)	4384
	Categorical [Ref. Quartile 1)				
	Quartile 2	-1.1074	0.2131	(-2.8522, 0.6375)	4386
	Quartile 3	-0.4932	0.5836	(-2.2594, 1.273)	4386
	Quartile 4	-0.4686	0.6009	(-2.2269, 1.2896)	4386
BC	Linear	0.0084	0.7706	(-0.0481, 0.0649)	3967
	Log linear	-0.2181	0.5048	(-0.86, 0.4238)	3967
	Categorical [Ref. Quartile 1)				
	Quartile 2	-1.2889	0.1745	(-3.1508, 0.573)	3967
	Quartile 3	0.0841	0.9289	(-1.7666, 1.9348)	3967
	Quartile 4	-1.2782	0.1729	(-3.1181, 0.5617)	3967
CO	Linear	-0.0046	0.927	(-0.103, 0.0938)	4357
	Log linear	-0.2803	0.2227	(-0.7313, 0.1707)	4356
	Categorical [Ref. Quartile 1)				
	Quartile 2	-2.0107	0.026	(-3.7805, -0.2408)	4353
	Quartile 3	0.0481	0.9574	(-1.7193, 1.8155)	4353
	Quartile 4	-1.5157	0.0931	(-3.2857, 0.2542)	4353
Diastolic Blood Pressure					
PM2.5	Linear	-0.0038	0.1327	(-0.0088, 0.0012)	4197
	Log linear	-0.6343	0.0193	(-1.1654, -0.1032)	4194
	Categorical [Ref. Quartile 1)				
	Quartile 2	-0.9091	0.2341	(-2.4082, 0.5899)	4196
	Quartile 3	-2.1263	0.0061	(-3.6437, -0.6089)	4196
	Quartile 4	-1.3251	0.0855	(-2.8356, 0.1855)	4196
BC	Linear	-0.0448	0.077	(-0.0945, 0.0049)	3821
	Log linear	-0.6638	0.0211	(-1.2274, -0.1002)	3818
	Categorical [Ref. Quartile 1)				
	Quartile 2	-0.3717	0.6557	(-2.0084, 1.265)	3820
	Quartile 3	-1.4124	0.0887	(-3.0392, 0.2144)	3820
	Quartile 4	-1.9757	0.0167	(-3.5931, -0.3584)	3820
CO	Linear	0.0141	0.7423	(-0.07, 0.0982)	4161
	Log linear	-0.3945	0.0444	(-0.7792, -0.0099)	4157
	Categorical [Ref. Quartile 1)				
	Quartile 2	-2.3678	0.0022	(-3.8776, -0.858)	4155
	Quartile 3	-1.2191	0.1128	(-2.7268, 0.2886)	4155
	Quartile 4	-1.8532	0.0162	(-3.3631, -0.3434)	4155

Note: *All models controlled for gestational age at baseline, BMI, and mother's age, physical activity, time of the BP measurement, household food insecurity, mother's diet diversity and season.*

Table S5b. Adjusted association between HAP and PP/MAP in Peru

	Model Type	Estimate	p-value	95% CI	AIC
Pulse Pressure					
PM2.5	Linear	0.0052	0.038	(0.0003, 0.0101)	4174
	Log linear	0.6109	0.0219	(0.0886, 1.1331)	4173
	Categorical [Ref. Quartile 1)				
	Quartile 2	-0.1983	0.7918	(-1.6726, 1.2761)	4175
	Quartile 3	1.6331	0.032	(0.1408, 3.1254)	4175
	Quartile 4	0.8564	0.258	(-0.6292, 2.342)	4175
BC	Linear	0.0532	0.0288	(0.0055, 0.1009)	3774
	Log linear	0.4457	0.1075	(-0.0974, 0.9888)	3776
	Categorical [Ref. Quartile 1)				
	Quartile 2	-0.9171	0.2515	(-2.4866, 0.6524)	3773
	Quartile 3	1.4965	0.06	(-0.0635, 3.0566)	3773
	Quartile 4	0.6975	0.3774	(-0.8534, 2.2484)	3773
CO	Linear	-0.0187	0.6552	(-0.1008, 0.0634)	4131
	Log linear	0.1142	0.5517	(-0.2624, 0.4908)	4131
	Categorical [Ref. Quartile 1)				
	Quartile 2	0.3571	0.6363	(-1.1255, 1.8398)	4132
	Quartile 3	1.2671	0.0933	(-0.2134, 2.7477)	4132
	Quartile 4	0.3375	0.655	(-1.1452, 1.8202)	4132
Mean Arterial Pressure					
PM2.5	Linear	-0.0021	0.3851	(-0.0068, 0.0026)	4132
	Log linear	-0.4307	0.0942	(-0.9353, 0.0739)	4130
	Categorical [Ref. Quartile 1)				
	Quartile 2	-0.9752	0.1794	(-2.4, 0.4496)	4132
	Quartile 3	-1.5819	0.0316	(-3.0241, -0.1398)	4132
	Quartile 4	-1.0396	0.1555	(-2.4753, 0.3961)	4132
BC	Linear	-0.0271	0.2577	(-0.074, 0.0199)	3756
	Log linear	-0.5153	0.0579	(-1.0478, 0.0172)	3754
	Categorical [Ref. Quartile 1)				
	Quartile 2	-0.6774	0.3901	(-2.2247, 0.8698)	3756
	Quartile 3	-0.9136	0.2438	(-2.4515, 0.6243)	3756
	Quartile 4	-1.7432	0.0255	(-3.2722, -0.2143)	3756
CO	Linear	0.0079	0.8476	(-0.0724, 0.0882)	4103
	Log linear	-0.3565	0.0572	(-0.7238, 0.0109)	4100
	Categorical [Ref. Quartile 1)				
	Quartile 2	-2.2488	0.0023	(-3.6894, -0.8082)	4096
	Quartile 3	-0.7967	0.2772	(-2.2353, 0.6419)	4096
	Quartile 4	-1.7407	0.018	(-3.1814, -0.3001)	4096

Note: *All models controlled for gestational age at baseline, BMI, and mother's age, physical activity, time of the BP measurement, household food insecurity, mother's diet diversity and season.*

Table S6a. Adjusted association between HAP and SBP/DBP in Rwanda

	Model Type	Estimate	p-value	95% CI	AIC
Systolic Blood Pressure					
PM2.5	Linear	-0.0062	0.1083	(-0.0138, 0.0014)	5047
	Log linear	-0.3295	0.5432	(-1.3931, 0.7341)	5049
	Categorical [Ref. Quartile 1)				
	Quartile 2	0.125	0.9068	(-1.9704, 2.2203)	5054
	Quartile 3	-0.1623	0.881	(-2.2906, 1.966)	5054
	Quartile 4	-0.0116	0.9917	(-2.1801, 2.1569)	5054
BC	Linear	0.0735	0.161	(-0.0294, 0.1764)	4017
	Log linear	1.0388	0.1603	(-0.4127, 2.4903)	4017
	Categorical [Ref. Quartile 1)				
	Quartile 2	-1.899	0.1048	(-4.1953, 0.3973)	4009
	Quartile 3	-2.788	0.0245	(-5.2163, -0.3596)	4009
	Quartile 4	1.0233	0.4081	(-1.4046, 3.4511)	4009
CO	Linear	-0.0626	0.4622	(-0.2298, 0.1045)	4984
	Log linear	0.0652	0.8124	(-0.4742, 0.6047)	4985
	Categorical [Ref. Quartile 1)				
	Quartile 2	1.2048	0.2363	(-0.791, 3.2006)	4986
	Quartile 3	1.3867	0.1704	(-0.5975, 3.3708)	4986
	Quartile 4	0.2918	0.7739	(-1.7018, 2.2855)	4986
Diastolic Blood Pressure					
PM2.5	Linear	-0.0077	0.0099	(-0.0136, -0.0019)	4694
	Log linear	-0.9896	0.0184	(-1.8119, -0.1674)	4695
	Categorical [Ref. Quartile 1)				
	Quartile 2	0.019	0.9817	(-1.6035, 1.6415)	4701
	Quartile 3	-0.9659	0.2502	(-2.614, 0.6821)	4701
	Quartile 4	-1.076	0.2088	(-2.7552, 0.6032)	4701
BC	Linear	-0.0534	0.1786	(-0.1314, 0.0245)	3713
	Log linear	-0.8706	0.1201	(-1.9692, 0.228)	3713
	Categorical [Ref. Quartile 1)				
	Quartile 2	-1.5368	0.0851	(-3.2869, 0.2133)	3712
	Quartile 3	-2.3581	0.0126	(-4.2089, -0.5074)	3712
	Quartile 4	-1.844	0.0508	(-3.6944, 0.0063)	3712
CO	Linear	0.0889	0.183	(-0.042, 0.2198)	4651
	Log linear	0.1331	0.5365	(-0.2896, 0.5559)	4652
	Categorical [Ref. Quartile 1)				
	Quartile 2	0.5553	0.4867	(-1.0114, 2.1221)	4656
	Quartile 3	0.1787	0.8218	(-1.3789, 1.7363)	4656
	Quartile 4	0.4048	0.6117	(-1.1603, 1.9699)	4656

Note: *All models controlled for gestational age at baseline, BMI, and mother's age, nulliparity, mother's education, physical activity, time of the BP measurement, household food insecurity and smoker at home.*

Table S6b. Adjusted association between HAP and PP/MAP in Rwanda

	Model Type	Estimate	p-value	95% CI	AIC
Pulse Pressure					
PM2.5	Linear	0.0015	0.6002	(-0.0042, 0.0072)	4655
	Log linear	0.6602	0.1044	(-0.1371, 1.4574)	4652
	Categorical [Ref. Quartile 1)				
	Quartile 2	0.106	0.8946	(-1.4645, 1.6764)	4656
	Quartile 3	0.8036	0.3229	(-0.7915, 2.3988)	4656
	Quartile 4	1.0645	0.1989	(-0.5608, 2.6898)	4656
BC	Linear	0.127	0.0012	(0.0501, 0.2038)	3698
	Log linear	1.9094	6.00E-04	(0.8268, 2.992)	3697
	Categorical [Ref. Quartile 1)				
	Quartile 2	-0.3623	0.6791	(-2.0817, 1.3572)	3693
	Quartile 3	-0.4299	0.6425	(-2.2482, 1.3885)	3693
	Quartile 4	2.8673	0.002	(1.0493, 4.6853)	3693
CO	Linear	-0.1515	0.0187	(-0.2777, -0.0253)	4601
	Log linear	-0.0679	0.7444	(-0.4767, 0.3409)	4606
	Categorical [Ref. Quartile 1)				
	Quartile 2	0.6494	0.399	(-0.8617, 2.1605)	4606
	Quartile 3	1.208	0.1148	(-0.2943, 2.7102)	4606
	Quartile 4	-0.113	0.8832	(-1.6225, 1.3965)	4606
Mean Arterial Pressure					
PM2.5	Linear	-0.0072	0.0166	(-0.0131, -0.0013)	4703
	Log linear	-0.7696	0.0686	(-1.5982, 0.059)	4705
	Categorical [Ref. Quartile 1)				
	Quartile 2	0.0543	0.948	(-1.5798, 1.6885)	4711
	Quartile 3	-0.6981	0.4092	(-2.3579, 0.9618)	4711
	Quartile 4	-0.7212	0.4027	(-2.4124, 0.97)	4711
BC	Linear	-0.0111	0.7828	(-0.0903, 0.068)	3730
	Log linear	-0.2341	0.6805	(-1.3506, 0.8823)	3730
	Categorical [Ref. Quartile 1)				
	Quartile 2	-1.6575	0.0668	(-3.4303, 0.1152)	3726
	Quartile 3	-2.5014	0.009	(-4.3761, -0.6267)	3726
	Quartile 4	-0.8883	0.3523	(-2.7626, 0.986)	3726
CO	Linear	0.0384	0.5659	(-0.0928, 0.1695)	4653
	Log linear	0.1105	0.6082	(-0.3126, 0.5336)	4653
	Categorical [Ref. Quartile 1)				
	Quartile 2	0.7718	0.334	(-0.7956, 2.3393)	4656
	Quartile 3	0.5814	0.4641	(-0.9769, 2.1396)	4656
	Quartile 4	0.3672	0.6454	(-1.1986, 1.9329)	4656

Note: *All models controlled for gestational age at baseline, BMI, and mother's age, nulliparity, mother's education, physical activity, time of the BP measurement, household food insecurity and smoker at home.*

Table S7. Trial-wide association between HAP exposure and blood pressure parameters (without the highest and lowest 2.5% of the exposure samples)

	$PM_{2.5}$		BC		CO	
	Estimate	95% CI	Estimate	95% CI	Estimate	95% CI
Systolic Blood Pressure						
Log linear	0.20	(-0.24, 0.65)	0.19	(-0.54, 0.92)	0.01	(-0.33, 0.36)
Categorical [Ref. Quartile 1]						
Quartile 2	0.11	(-0.84, 1.05)	-0.67	(-1.79, 0.44)	-0.49	(-2.33, 1.34)
Quartile 3	0.26	(-0.69, 1.22)	-0.33	(-1.90, 1.25)	0.67	(-0.49, 1.84)
Quartile 4	0.50	(-0.48, 1.48)	0.13	(-1.26, 1.52)	-0.37	(-1.47, 0.74)
Diastolic Blood Pressure						
Log linear	-0.12	(-0.94, 0.70)	-0.25	(-1.28, 0.79)	-0.07	(-0.59, 0.45)
Categorical [Ref. Quartile 1]						
Quartile 2	0.00	(-0.77, 0.78)	-0.40	(-1.29, 0.49)	-0.70	(-2.29, 0.88)
Quartile 3	-0.29	(-1.78, 1.21)	-0.65	(-2.31, 1.01)	-0.12	(-1.43, 1.18)
Quartile 4	0.05	(-1.36, 1.46)	-0.74	(-2.50, 1.02)	-0.16	(-2.00, 1.69)
Pulse Pressure						
Log linear	0.38	(-0.01, 0.78)	0.55	(-0.33, 1.42)	0.07	(-0.14, 0.28)
Categorical [Ref. Quartile 1]						
Quartile 2	0.12	(-0.61, 0.85)	-0.31	(-1.08, 0.46)	0.14	(-0.56, 0.85)
Quartile 3	0.54	(-0.37, 1.44)	0.23	(-0.64, 1.11)	0.73	(0.02, 1.44)
Quartile 4	0.52	(-0.24, 1.28)	0.89	(-0.29, 2.07)	-0.13	(-0.89, 0.63)
Mean Arterial Pressure						
Log linear	0.00	(-0.69, 0.68)	-0.06	(-0.92, 0.80)	-0.05	(-0.51, 0.42)
Categorical [Ref. Quartile 1]						
Quartile 2	0.02	(-0.74, 0.78)	-0.50	(-1.44, 0.45)	-0.63	(-2.29, 1.03)
Quartile 3	-0.11	(-1.31, 1.09)	-0.56	(-2.14, 1.03)	0.13	(-1.09, 1.36)
Quartile 4	0.06	(-1.33, 1.45)	-0.43	(-1.98, 1.13)	-0.22	(-1.83, 1.40)

Notes:
1. All models controlled for gestational age at the BP measurement, BMI, and mother's age. Additional covariates are controlled for IRC-specific models (Guatemala: nulliparity, mother's education, physical activity, date of the BP measurement, mother's diet diversity and season; India: mother's education, household wealth, and season; Peru: physical activity, time of the BP measurement, household food insecurity, mother's diet diversity and season; Rwanda: nulliparity, mother's education, physical activity, time of the BP measurement, household food insecurity and smoker at home)
2. In log linear models, the coefficients indicate the increase in BP (mmHg) per a one unit increase in the log of exposure.
3. Shaded cells are fixed effects, unshaded are random effects, meta-analyses combining results across 4 IRCs

Table S8. Effect modification by gestational age at BP measurement, maternal age, BMI, physical activity, and presence of smoker at home for the association between HAP exposure and BP parameters based on log-linear exposure models in **Guatemala**

Interaction	Exposure	Estimate	SE	P-value	Estimate	SE	P-value
			**SBP**			**DBP**	
Gestational age at baseline BP measurement [Ref. <= Median]	$PM_{2.5}$	0.38	0.80	0.64	0.55	0.67	0.41
	BC	1.14	1.32	0.39	0.90	1.11	0.41
	CO	-0.57	0.49	0.24	-0.28	0.42	0.51

Maternal age [Ref. <= Median]	$PM_{2.5}$	0.04	0.81	0.96	0.68	0.68	0.31
	BC	-0.88	1.33	0.51	0.00	1.11	1.00
	CO	-0.20	0.49	0.68	0.12	0.42	0.78
Baseline BMI [Ref. Normal weight] (Coefficients in the order of 'Obese', 'Overweight', and 'Underweight')	$PM_{2.5}$	1.85	2.08	0.38	1.44	1.75	0.41
		-0.92	0.93	0.32	0.77	0.78	0.32
		4.45	5.38	0.41	6.60	4.51	0.14
	BC	2.94	3.16	0.35	1.74	2.64	0.51
		-0.81	1.45	0.58	0.96	1.22	0.43
		-10.34	30.04	0.73	-24.48	25.13	0.33
	CO	2.43	1.33	0.07	1.43	1.13	0.21
		-1.29	0.54	0.02	-0.32	0.46	0.49
		3.29	4.56	0.47	5.40	3.89	0.17
Baseline physical activity [Ref. <= Median]	$PM_{2.5}$	-0.13	0.80	0.88	0.51	0.67	0.45
	BC	0.16	1.35	0.91	-0.39	1.13	0.73
	CO	-0.10	0.49	0.84	0.12	0.42	0.78
Smoker present at home [Ref. No/NA]	$PM_{2.5}$	-2.28	1.92	0.24	-0.57	1.61	0.72
	BC	0.97	4.30	0.82	-2.08	3.60	0.56
	CO	-1.31	1.12	0.24	-0.28	0.95	0.77
			**PP**			**MAP**	
Gestational age at baseline BP measurement [Ref. <= Median]	$PM_{2.5}$	-0.17	0.56	0.76	0.50	0.67	0.46
	BC	0.24	0.95	0.80	0.98	1.09	0.37
	CO	-0.30	0.34	0.39	-0.38	0.41	0.36
Maternal age [Ref. <= Median]	$PM_{2.5}$	-0.64	0.56	0.25	0.47	0.67	0.48
	BC	-0.88	0.96	0.36	-0.29	1.10	0.79
	CO	-0.32	0.34	0.35	0.01	0.41	0.98
Baseline BMI [Ref. Normal weight] (Coefficients in the order of 'Obese', 'Overweight', and 'Underweight')	$PM_{2.5}$	0.41	1.45	0.78	1.58	1.74	0.36
		-1.68	0.65	0.01	0.20	0.77	0.79
		-2.15	3.75	0.57	5.88	4.48	0.19
	BC	1.20	2.27	0.60	2.14	2.61	0.41
		-1.76	1.04	0.09	0.37	1.20	0.76
		14.14	21.58	0.51	-19.77	24.86	0.43
	CO	1.00	0.93	0.28	1.76	1.12	0.11
		-0.96	0.38	0.01	-0.64	0.46	0.16
		-2.11	3.19	0.51	4.70	3.84	0.22
Baseline physical activity [Ref. <= Median]	$PM_{2.5}$	-0.64	0.56	0.26	0.30	0.67	0.65
	BC	0.55	0.97	0.57	-0.21	1.12	0.85
	CO	-0.21	0.34	0.53	0.04	0.41	0.91
Smoker present at home [Ref. No/NA]	$PM_{2.5}$	-1.71	1.34	0.20	-1.14	1.60	0.48
	BC	3.05	3.10	0.32	-1.06	3.56	0.77
	CO	-1.02	0.78	0.19	-0.62	0.94	0.51

Table S9. Effect modification by gestational age at BP measurement, maternal age, BMI, physical activity, and presence of smoker at home for the association between HAP exposure and BP parameters based on log-linear exposure models in **India**

Interaction	Exposure	Estimate	SE	P-value	Estimate	SE	P-value
			**SBP**			**DBP**	
Gestational age at baseline BP measurement [Ref. <= Median]	$PM_{2.5}$	0.73	0.83	0.38	0.68	0.68	0.31
	BC	-0.32	0.83	0.70	-0.05	0.67	0.95
	CO	0.21	0.41	0.62	0.19	0.34	0.57
Maternal age [Ref. <= Median]	$PM_{2.5}$	1.15	0.85	0.18	0.84	0.69	0.23
	BC	1.28	0.83	0.12	0.94	0.68	0.17
	CO	0.17	0.41	0.69	0.30	0.34	0.38
Baseline BMI [Ref. Normal weight] (Coefficients in the order of 'Obese', 'Overweight', and 'Underweight')	$PM_{2.5}$	7.39	3.21	0.02	2.95	2.60	0.26
		-0.48	1.96	0.81	-3.26	1.59	0.04
		0.15	0.89	0.87	0.21	0.72	0.77
	BC	14.23	5.16	0.01	5.87	4.22	0.16
		1.26	1.71	0.46	-1.96	1.40	0.16
		-0.08	0.89	0.93	0.16	0.73	0.82
	CO	1.45	1.68	0.39	0.63	1.39	0.65
		1.37	0.94	0.15	0.98	0.78	0.21
		-0.27	0.43	0.53	0.05	0.36	0.89
Baseline physical activity [Ref. <= Median]	$PM_{2.5}$	-0.01	0.83	0.99	0.00	0.68	1.00
	BC	0.98	0.83	0.24	0.13	0.68	0.84
	CO	0.52	0.42	0.21	0.24	0.34	0.49
Smoker present at home [Ref. No/NA]	$PM_{2.5}$	-0.84	0.89	0.35	-0.48	0.73	0.50
	BC	-0.33	0.90	0.71	-0.16	0.74	0.83
	CO	0.57	0.44	0.19	0.64	0.36	0.08
			**PP**			**MAP**	
Gestational age at baseline BP measurement [Ref. <= Median]	$PM_{2.5}$	0.05	0.69	0.95	0.70	0.65	0.29
	BC	-0.27	0.68	0.69	-0.14	0.65	0.83
	CO	0.01	0.34	0.97	0.20	0.33	0.55
Maternal age [Ref. <= Median]	$PM_{2.5}$	0.32	0.71	0.66	0.94	0.67	0.16
	BC	0.34	0.69	0.62	1.05	0.66	0.11
	CO	-0.14	0.34	0.69	0.26	0.33	0.44
Baseline BMI [Ref. Normal weight] (Coefficients in the order of 'Obese', 'Overweight', and 'Underweight')	$PM_{2.5}$	4.44	2.66	0.10	4.43	2.52	0.08
		2.78	1.62	0.09	-2.34	1.54	0.13
		-0.06	0.74	0.93	0.19	0.70	0.79
	BC	8.36	4.26	0.05	8.66	4.09	0.03
		3.21	1.41	0.02	-0.88	1.35	0.51
		-0.24	0.73	0.74	0.08	0.70	0.91
	CO	0.82	1.37	0.55	0.91	1.34	0.50
		0.38	0.77	0.62	1.11	0.75	0.14
		-0.32	0.35	0.36	-0.06	0.35	0.87
Baseline physical activity [Ref. <= Median]	$PM_{2.5}$	-0.01	0.69	0.99	0.00	0.65	1.00
	BC	0.85	0.69	0.22	0.42	0.66	0.53

	CO	0.28	0.34	0.40	0.33	0.33	0.32
Smoker present at home [Ref. No/NA]	$PM_{2.5}$	-0.36	0.74	0.63	-0.60	0.70	0.39
	BC	-0.17	0.75	0.81	-0.22	0.71	0.76
	CO	-0.07	0.36	0.85	0.62	0.35	0.08

Table S10. Effect modification by gestational age at BP measurement, maternal age, BMI, physical activity, and presence of smoker at home for the association between HAP exposure and BP parameters based on log-linear exposure models in **Peru**

Interaction	Exposure	Estimate	SE	P-value	Estimate	SE	P-value
			**SBP**			**DBP**	
Gestational age at baseline BP measurement [Ref. <= Median]	$PM_{2.5}$	0.80	0.63	0.21	-0.52	0.54	0.34
	BC	0.15	0.65	0.82	-0.70	0.57	0.22
	CO	-0.08	0.46	0.87	0.32	0.39	0.42
Maternal age [Ref. <= Median]	$PM_{2.5}$	-0.52	0.63	0.41	-1.44	0.54	0.01
	BC	-0.27	0.65	0.68	-1.38	0.57	0.02
	CO	-0.94	0.45	0.04	-0.77	0.39	0.05
Baseline BMI [Ref. Normal weight] (Coefficients in the order of 'Obese', 'Overweight', and 'Underweight')	$PM_{2.5}$	2.36	0.99	0.02	0.81	0.85	0.34
		1.05	0.67	0.12	0.14	0.58	0.81
		NA	NA	NA	NA	NA	NA
	BC	2.26	1.05	0.03	0.88	0.93	0.34
		0.99	0.70	0.16	0.26	0.61	0.67
		NA	NA	NA	NA	NA	NA
	CO	1.15	0.72	0.11	0.78	0.61	0.21
		-0.34	0.49	0.48	0.12	0.42	0.78
		NA	NA	NA	NA	NA	NA
Baseline physical activity [Ref. <= Median]	$PM_{2.5}$	0.10	0.63	0.87	-0.59	0.54	0.27
	BC	-0.30	0.65	0.64	-0.84	0.57	0.14
	CO	-0.16	0.45	0.72	-0.60	0.39	0.12
Smoker present at home [Ref. No/NA]	$PM_{2.5}$	0.87	3.87	0.82	-1.23	3.33	0.71
	BC	-2.56	4.75	0.59	-4.95	4.17	0.24
	CO	2.84	6.41	0.66	3.04	5.46	0.58
			**PP**			**MAP**	
Gestational age at baseline BP measurement [Ref. <= Median]	$PM_{2.5}$	1.32	0.53	0.01	-0.08	0.51	0.87
	BC	0.85	0.55	0.12	-0.42	0.54	0.44
	CO	-0.39	0.38	0.31	0.19	0.38	0.62
Maternal age [Ref. <= Median]	$PM_{2.5}$	0.92	0.53	0.08	-1.13	0.51	0.03
	BC	1.11	0.55	0.04	-1.01	0.54	0.06
	CO	-0.17	0.38	0.65	-0.83	0.37	0.03
Baseline BMI [Ref. Normal weight] (Coefficients in the order of 'Obese', 'Overweight', and 'Underweight')	$PM_{2.5}$	1.55	0.83	0.06	1.32	0.81	0.10
		0.91	0.57	0.11	0.44	0.55	0.42
		NA	NA	NA	NA	NA	NA
	BC	1.38	0.89	0.12	1.34	0.87	0.13
		0.73	0.59	0.22	0.50	0.58	0.39

		NA	NA	NA	NA	NA	NA
	CO	0.37	0.60	0.53	0.90	0.59	0.12
		-0.46	0.40	0.26	-0.04	0.40	0.93
		NA	NA	NA	NA	NA	NA
Baseline physical activity [Ref. <= Median]	$PM_{2.5}$	0.70	0.53	0.19	-0.36	0.51	0.48
	BC	0.53	0.55	0.33	-0.66	0.54	0.22
	CO	0.44	0.38	0.25	-0.45	0.37	0.22
Smoker present at home [Ref. No/NA]	$PM_{2.5}$	2.09	3.27	0.52	-0.53	3.16	0.87
	BC	2.38	4.02	0.55	-4.15	3.94	0.29
	CO	-0.20	5.35	0.97	2.97	5.22	0.57

Table S11. Effect modification by gestational age at BP measurement, maternal age, BMI, physical activity, and presence of smoker at home for the association between HAP exposure and BP parameters based on log-linear exposure models in **Rwanda**

Interaction	Exposure	Estimate	SE	P-value	Estimate	SE	P-value
			**SBP**			**DBP**	
Gestational age at baseline BP measurement [Ref. <= Median]	$PM_{2.5}$	-0.14	0.99	0.88	0.82	0.76	0.28
	BC	-0.48	1.33	0.72	0.28	1.01	0.78
	CO	0.35	0.55	0.52	0.09	0.43	0.84
Maternal age [Ref. <= Median]	$PM_{2.5}$	-1.02	1.00	0.31	-0.30	0.77	0.69
	BC	-0.88	1.35	0.51	-0.66	1.02	0.52
	CO	-0.03	0.55	0.96	-0.78	0.43	0.07
Baseline BMI [Ref. Normal weight] (Coefficients in the order of 'Obese', 'Overweight', and 'Underweight')	$PM_{2.5}$	2.88	2.31	0.21	2.01	1.79	0.26
		-0.24	1.22	0.85	-0.47	0.95	0.62
		-3.06	4.08	0.45	0.90	3.16	0.78
	BC	1.83	3.03	0.55	1.88	2.29	0.41
		-0.86	1.68	0.61	-1.10	1.27	0.39
		0.15	5.35	0.98	-0.68	4.04	0.87
	CO	-0.66	1.02	0.52	-0.52	0.80	0.51
		0.90	0.69	0.19	0.67	0.54	0.21
		-1.96	2.18	0.37	-0.49	1.71	0.77
Baseline physical activity [Ref. <= Median]	$PM_{2.5}$	-0.05	1.05	0.96	-0.48	0.81	0.56
	BC	-1.29	1.42	0.36	-1.58	1.07	0.14
	CO	-0.60	0.55	0.28	-0.32	0.43	0.46
Smoker present at home [Ref. No/NA]	$PM_{2.5}$	1.17	2.36	0.62	0.30	1.83	0.87
	BC	0.11	2.87	0.97	0.64	2.17	0.77
	CO	-0.80	1.83	0.66	-0.62	1.43	0.66
			**PP**			**MAP**	
Gestational age at baseline BP measurement [Ref. <= Median]	$PM_{2.5}$	-0.96	0.74	0.19	0.50	0.77	0.52
	BC	-0.76	0.99	0.44	0.03	1.02	0.98
	CO	0.27	0.41	0.52	0.18	0.43	0.68
Maternal age [Ref. <= Median]	$PM_{2.5}$	-0.71	0.75	0.34	-0.54	0.78	0.49
	BC	-0.22	1.00	0.83	-0.73	1.03	0.48
	CO	0.75	0.42	0.07	-0.53	0.43	0.22

Baseline BMI [Ref. Normal weight] (Coefficients in the order of 'Obese', 'Overweight', and 'Underweight')	$PM_{2.5}$	0.87	1.71	0.61	2.30	1.81	0.20
		0.23	0.91	0.80	-0.39	0.96	0.68
		-3.95	3.02	0.19	-0.42	3.19	0.90
	BC	-0.05	2.24	0.98	1.86	2.33	0.42
		0.23	1.24	0.85	-1.02	1.29	0.43
		0.83	3.96	0.83	-0.41	4.12	0.92
	CO	-0.14	0.77	0.86	-0.57	0.80	0.48
		0.23	0.52	0.65	0.75	0.54	0.17
		-1.47	1.64	0.37	-0.98	1.71	0.57
Baseline physical activity [Ref. <= Median]	$PM_{2.5}$	0.43	0.79	0.59	-0.33	0.82	0.68
	BC	0.29	1.06	0.78	-1.48	1.09	0.17
	CO	-0.29	0.42	0.49	-0.41	0.43	0.34
Smoker present at home [Ref. No/NA]	$PM_{2.5}$	0.87	1.77	0.62	0.59	1.84	0.75
	BC	-0.53	2.14	0.81	0.46	2.21	0.83
	CO	-0.18	1.38	0.90	-0.68	1.43	0.64

Table S12. Summary of SBP, DBP and selected baseline characteristics in population with and without missing exposures to $PM_{2.5}$, BC, and CO

	**$PM_{2.5}$**		**BC**		**CO**	
	Missing	Non-missing	Missing	Non-missing	Missing	Non-missing
SBP (mmHg), Mean (SD)	103.6 (8.9)*	105.0 (9.8)*	104.6 (10.0)	104.9 (9.6)	103.5 (9.6)*	105.0 (9.7)*
DBP (mmHg), Mean (SD)	60.3 (7.6)	60.8 (7.8)	60.6 (8.0)	60.8 (7.8)	60.1 (7.7)	60.8 (7.8)
Maternal age (years), Mean (SD)	25.2 (4.4)	25.4 (4.5)	25.6 (4.4)	25.3 (4.5)	25.4 (4.3)	25.4 (4.5)
BMI (kg/m2), Mean (SD)	23.3 (4.2)	23.2 (4.0)	23.6 (4.2)*	23.1 (4.0)*	24.1 (4.0)*	23.1 (4.1)*
Nulliparous, N (%)						
Yes	134 (36%)	1094 (29%)	219 (34%)	1009 (40%)	126 (40%)	1102 (38%)
No	238 (64%)	1718 (61%)	434 (66%)	1522 (60%)	191 (60%)	1765 (62%)
Missing	0	6 (<1%)	1 (<1%)	5 (<1%)	1 (<1%)	5 (<1%)
Mother's Education, N (%)			*	*		
No formal education or Primary school incomplete	127 (34%)	912 (32%)	234 (36%)	805 (32%)	63 (20%)	976 (34%)
Primary school complete or Secondary school incomplete	122 (33%)	967 (34%)	229 (35%)	860 (34%)	109 (34%)	980 (34%)
Secondary school complete or Vocational or Some college or university	122 (33%)	939 (33%)	190 (29%)	871 (34%)	146 (46%)	915 (32%)
Missing	1 (<1%)	0	1 (<1%)	0	0	1 (<1%)

Note:

*1. * indicates statistical significance at $\alpha = 0.05$. T-tests were performed for continuous variable and chi-square tests were performed for categorical variable.*

2. Summary based on the 3190 pregnant women included in the analysis.

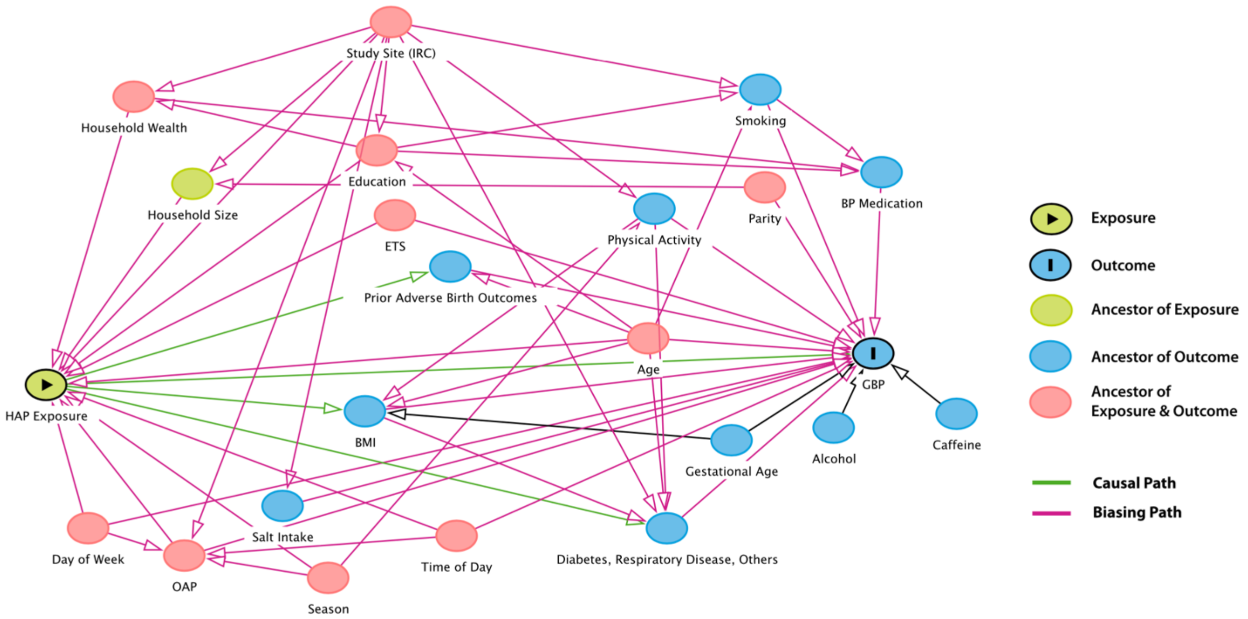

Figure S1. Directed Acyclic Graph (DAG) for the association between household air pollution (HAP) exposure from solid fuels and gestation blood pressure (GBP)

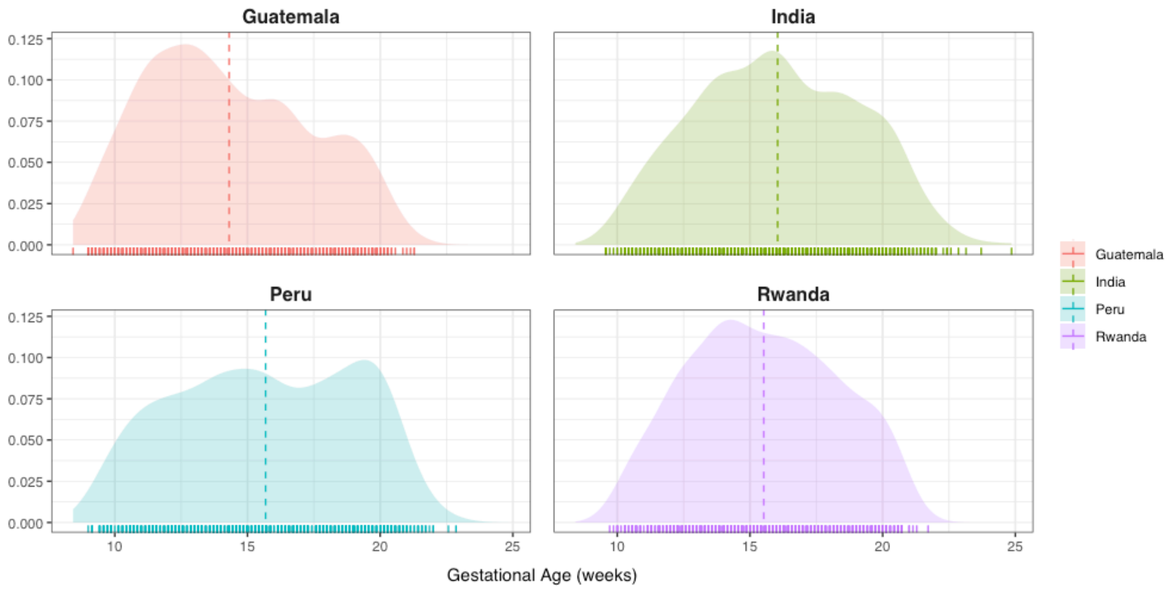

Figure S2. Distributions of gestational age at baseline. Solid lines along the x-axes are individual data points. Dashed lines are mean values.

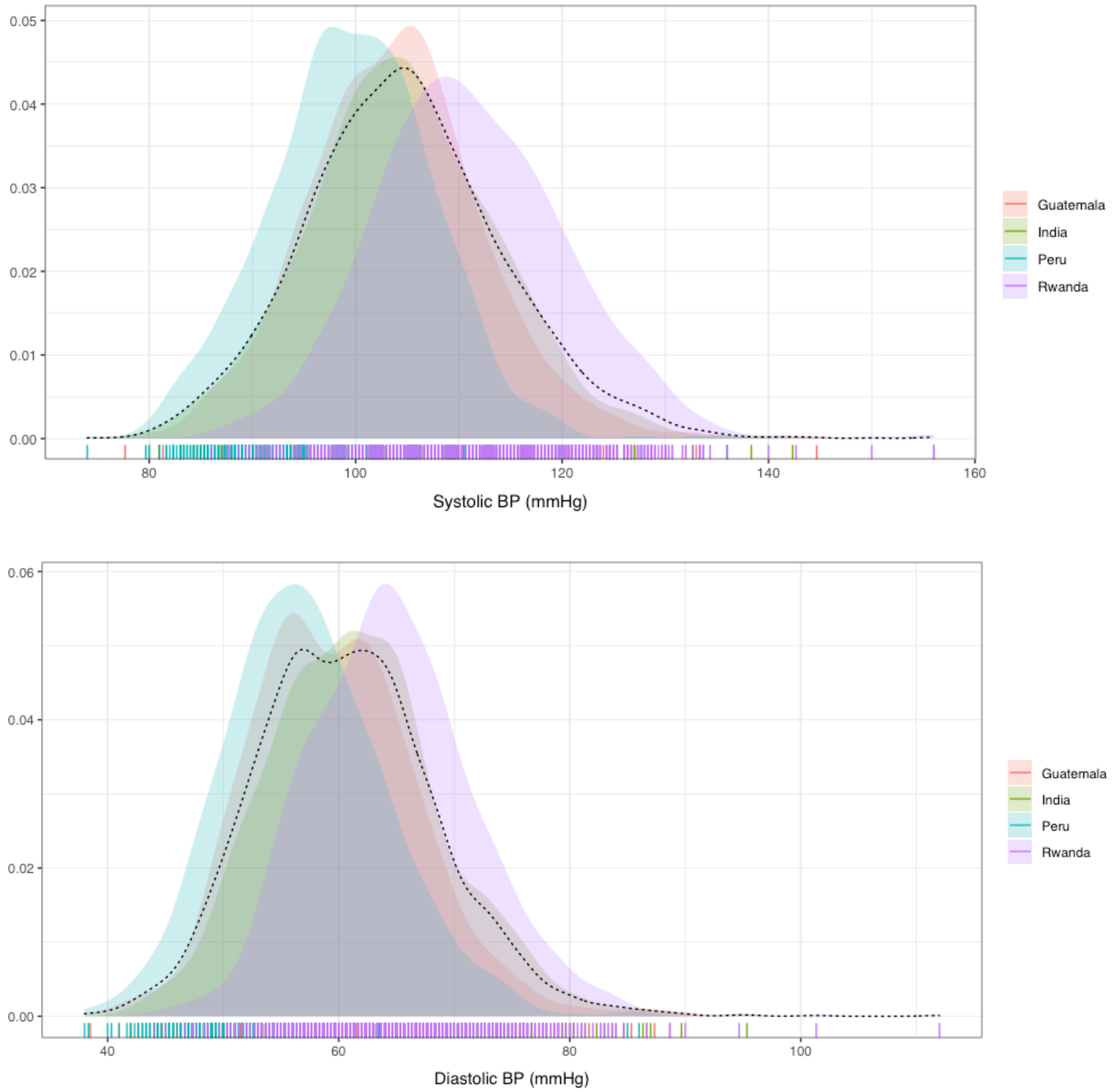

Figure S3. Distributions of systolic (top) and diastolic (bottom) blood pressure at baseline. Filled density plots are individual IRC distributions; the dotted line with no fill is the HAPIN-wide distribution. Individual data points are shown as bars along the x-axes.

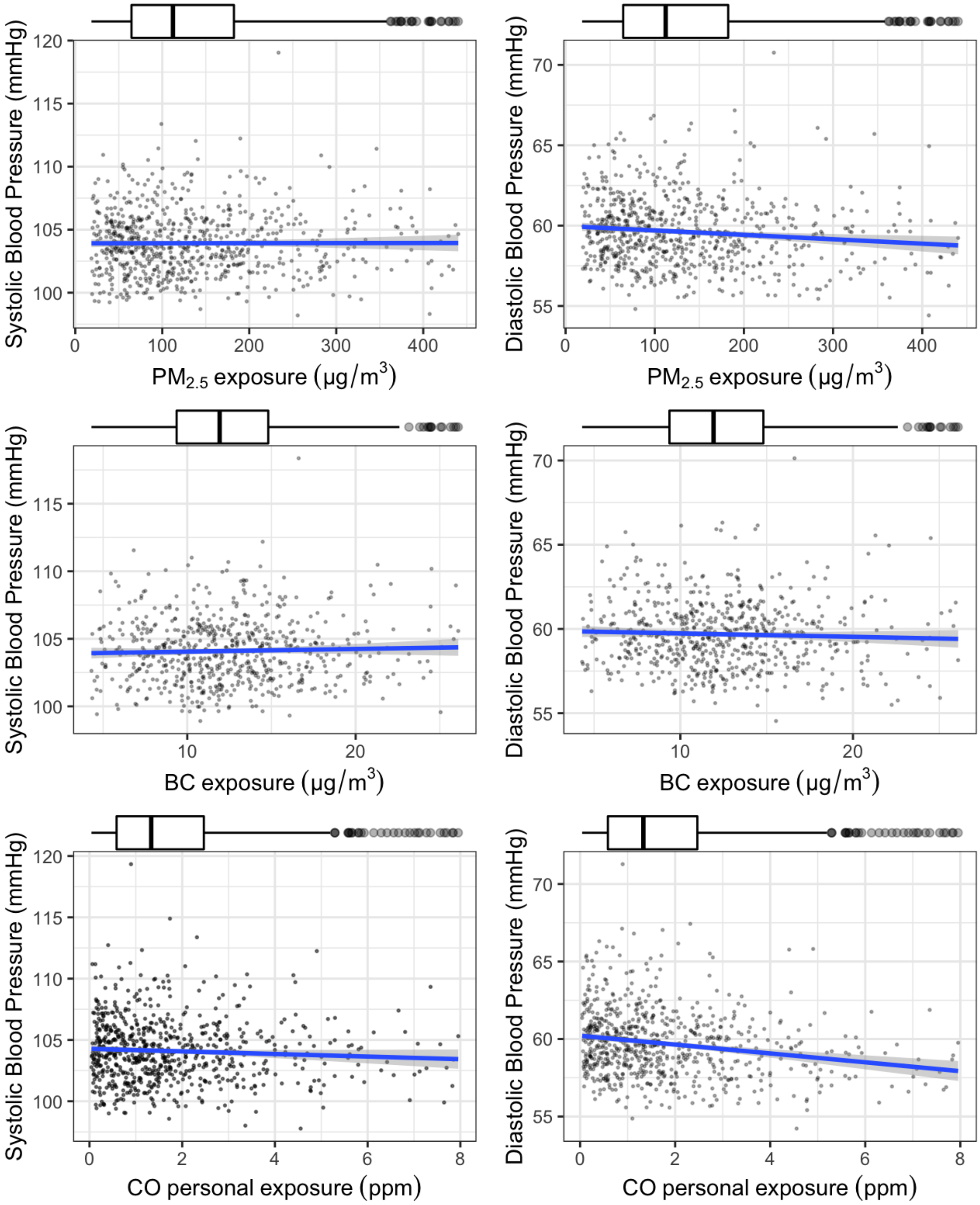

Figure S4. HAP-GBP association (blue line) and 95% confidence intervals (shade) in **Guatemala** generated from generalized additive models (GAMs) controlling for gestational age at the BP measurement, BMI, and mother's age, nulliparity, mother's education, physical activity, date of the BP measurement (weekday vs. weekend), mother's diet diversity and season (dry vs. rainy). All plots are showing 95% (after removing the highest and lowest 2.5%) of exposure the data for each household air pollutant.

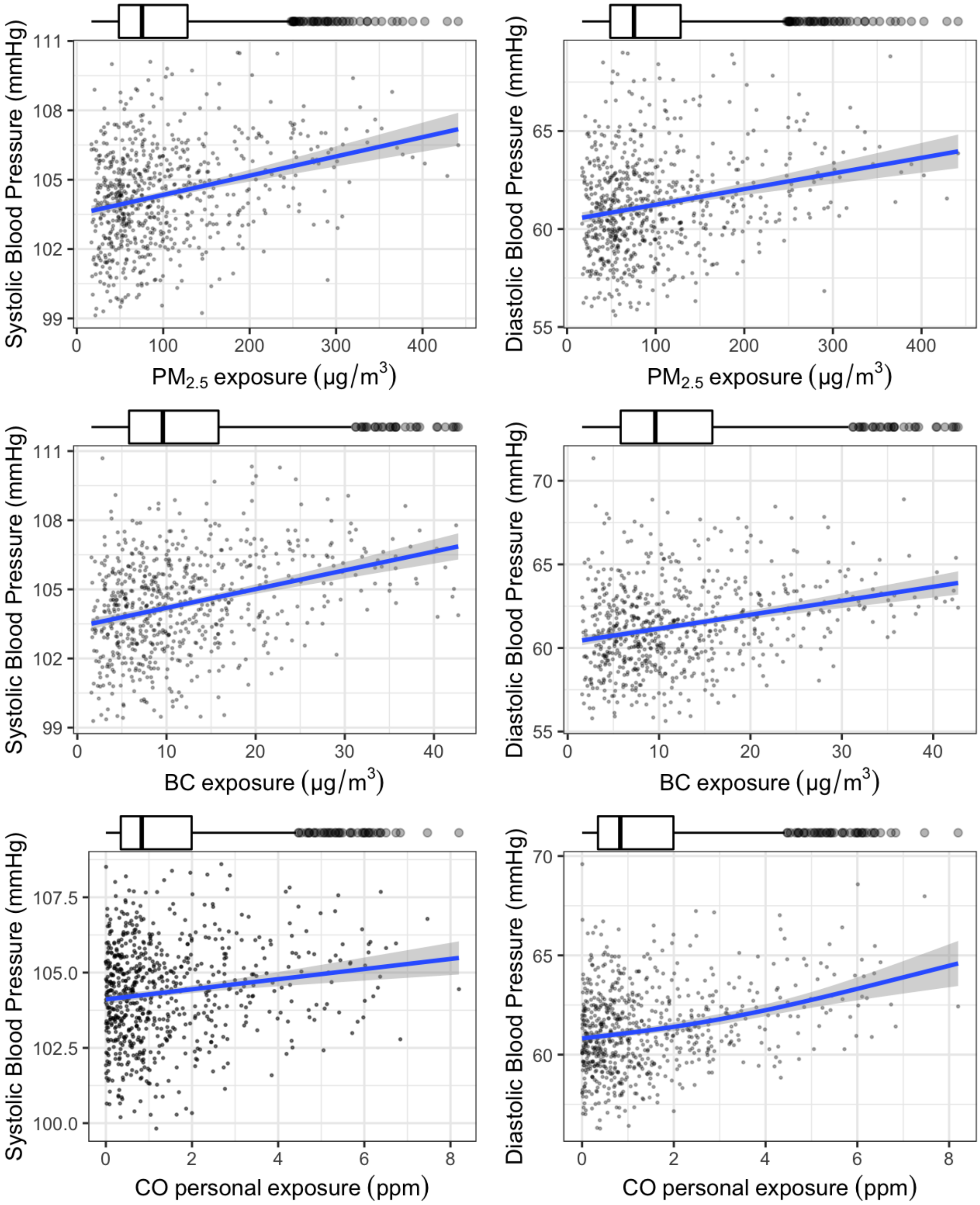

Figure S5. HAP-GBP association (blue line) and 95% confidence intervals (shade) in **India** generated from generalized additive models (GAMs) controlling for gestational age at baseline, BMI, mother's age, mother's education, household wealth, and season (winter, summer, and monsoon). All plots are showing 95% (after removing the highest and lowest 2.5%) of exposure the data for each household air pollutant.

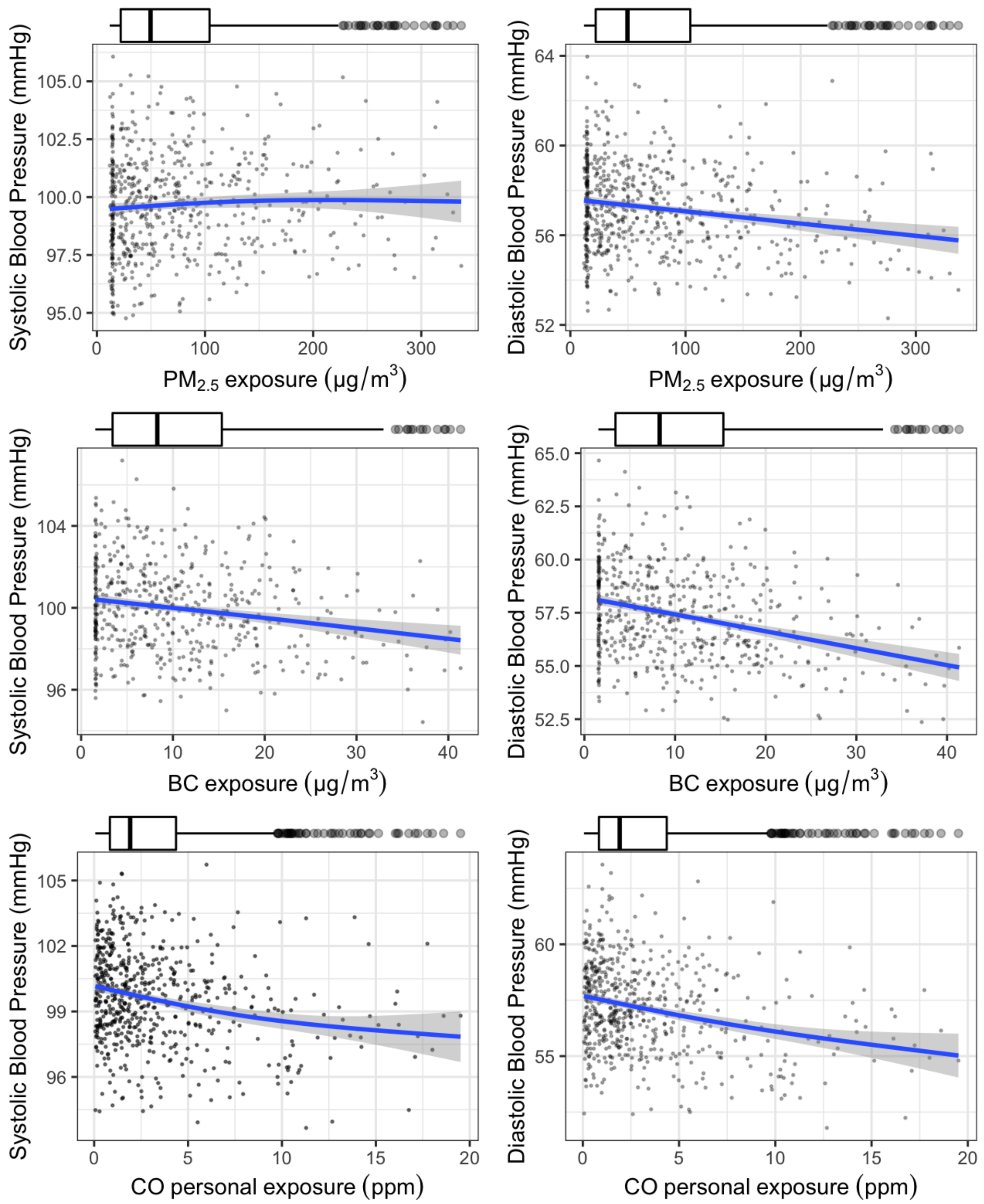

Figure S6. HAP-GBP association (blue line) and 95% confidence intervals (shade) in **Peru** generated from generalized additive models (GAMs) controlling for gestational age at baseline, BMI, and mother's age, physical activity, time of the BP measurement (morning vs. afternoon), household food insecurity, mother's diet diversity and season (dry vs. rainy). All plots are showing 95% (after removing the highest and lowest 2.5%) of exposure the data for each household air pollutant.

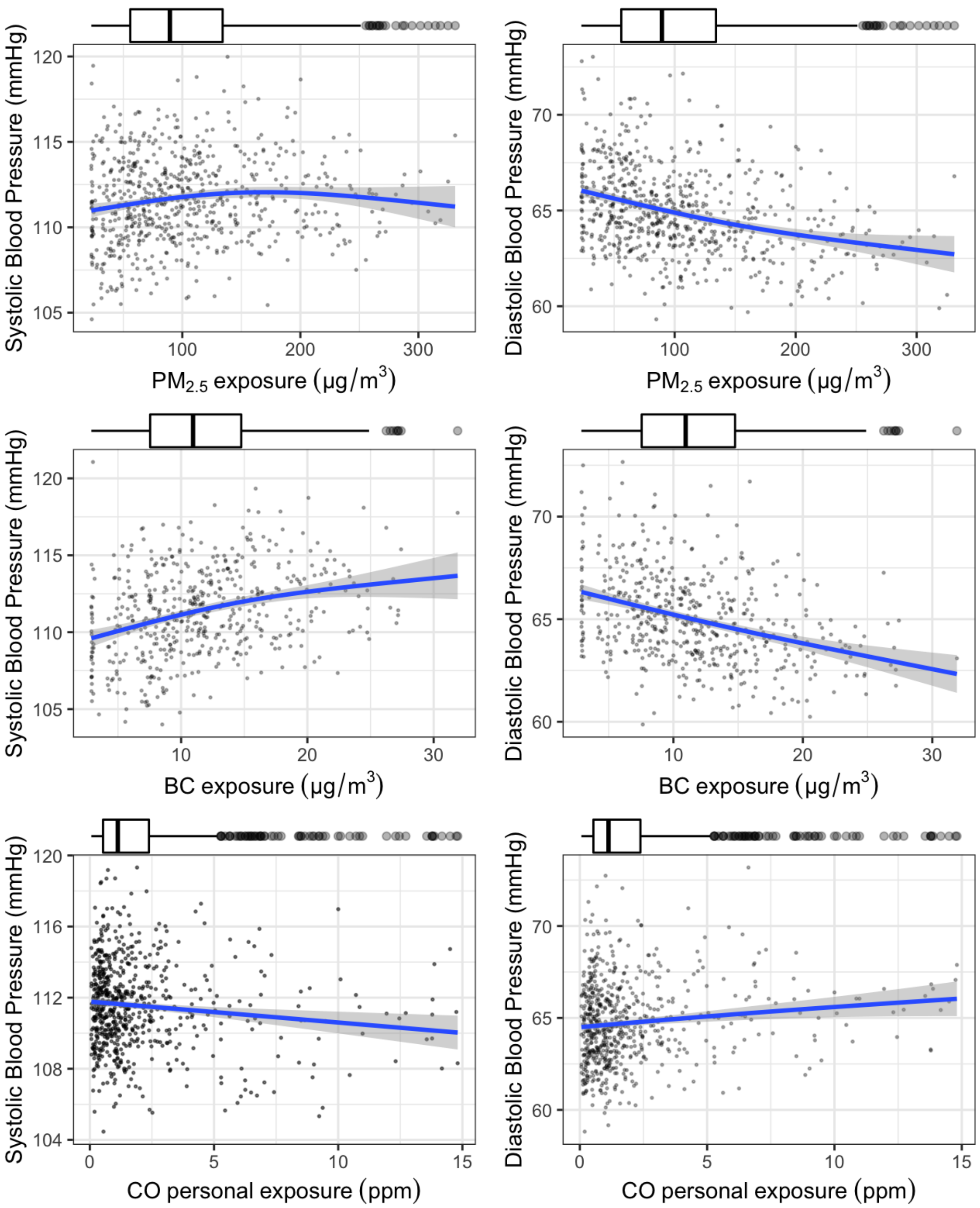

Figure S7. HAP-GBP association (blue line) and 95% confidence intervals (shade) in **Rwanda** generated from generalized additive models (GAMs) controlling for gestational age at baseline, BMI, and mother's age, nulliparity, mother's education, physical activity, time of the BP measurement (morning vs. afternoon), household food insecurity and smoker at home. All plots are showing 95% (after removing the highest and lowest 2.5%) of exposure the data for each household air pollutant.
